# Pseudorabies virus usurps non-muscle myosin heavy chain IIA to dampen viral DNA recognition by cGAS for antagonism of host antiviral innate immunity

**DOI:** 10.1128/jvi.00483-24

**Published:** 2024-04-19

**Authors:** Yingqi Liu, Yidan Qin, Bingbing Yang, Hongmei Zheng, Songlin Qiao, Zhong Luo, Rui Li

**Affiliations:** 1 School of Life Sciences, Chongqing University, Chongqing, China; 2 Key Laboratory of Animal Immunology of the Ministry of Agriculture, Henan Provincial Key Laboratory of Animal Immunology, Henan Academy of Agricultural Sciences, Zhengzhou, Henan, China; Lerner Research Institute, Cleveland Clinic, Cleveland, Ohio, USA

**Keywords:** PRV, NMHC-IIA, innate immunity, cGAS, PARP1, DNA recognition

## Abstract

Alphaherpesvirus pseudorabies virus (PRV) causes severe economic losses to the global pig industry and has garnered increasing attention due to its broad host range including humans. PRV has developed a variety of strategies to antagonize host antiviral innate immunity. However, the underlying mechanisms have not been fully elucidated. In our previous work, we demonstrated that non-muscle myosin heavy chain IIA (NMHC-IIA), a multifunctional cytoskeleton protein, attenuates innate immune responses triggered by RNA viruses. In the current study, we reported a previously unrecognized role of NMHC-IIA in counteracting PRV-induced cyclic GMP-AMP synthase (cGAS)-dependent type I interferon (IFN-I) production. Mechanistically, PRV infection led to an elevation of NMHC-IIA, strengthening the interaction between poly (ADP-ribose) polymerase 1 (PARP1) and cGAS. This interaction impeded cGAS recognition of PRV DNA and hindered downstream signaling activation. Conversely, inhibition of NMHC-IIA by Blebbistatin triggered innate immune responses and enhanced resistance to PRV proliferation both *in vitro* and *in vivo*. Taken together, our findings unveil that PRV utilizes NMHC-IIA to antagonize host antiviral immune responses via impairing DNA sensing by cGAS. This in-depth understanding of PRV immunosuppression not only provides insights for potential PRV treatment strategies but also highlights NMHC-IIA as a versatile immunosuppressive regulator usurped by both DNA and RNA viruses. Consequently, NMHC-IIA holds promise as a target for the development of broad-spectrum antiviral drugs.

**IMPORTANCE:**

Cyclic GMP-AMP synthase (cGAS)-stimulator of interferon genes (STING) axis plays a vital role in counteracting alphaherpesvirus infections. Alphaherpesviruses exploit various strategies for antagonizing cGAS-STING-mediated antiviral immune responses. However, limited examples of pseudorabies virus (PRV)-caused immunosuppression have been documented. Our findings reveal a novel role of non-muscle myosin heavy chain IIA (NMHC-IIA) in suppressing PRV-triggered innate immune responses to facilitate viral propagation both *in vitro* and *in vivo*. In detail, NMHC-IIA recruits poly (ADP-ribose) polymerase 1 (PARP1) to augment its interaction with cGAS, which impairs cGAS recognition of PRV DNA. Building on our previous demonstration of NMHC-IIA’s immunosuppressive role during RNA virus infections, these findings indicate that NMHC-IIA acts as a broad-spectrum suppressor of host antiviral innate immunity in response to both DNA and RNA viruses. Therefore, NMHC-IIA will be a promising target for the development of comprehensive antiviral strategies.

## INTRODUCTION

To cope with DNA virus infections, cyclic GMP-AMP synthase (cGAS) recognizes the viral DNA and subsequently activates the stimulator of interferon genes (STING)-tank binding kinase 1 (TBK1) signaling for interferon regulatory factor (IRF) 3-mediated production of interferon (IFN)-α/β and inflammatory cytokines (1, 2). IFN-α/β are potent host antiviral factors in the induction of interferon-stimulated genes (ISGs) to facilitate the establishment of the cellular antiviral state (3). However, DNA viruses have evolved diverse strategies to antagonize cGAS-STING-mediated antiviral responses (3–5).

Pseudorabies virus (PRV) belongs to the member of alphaherpesvirus subfamily and infects multiple vertebrates including pigs, wild boars, sheep, goats, bears, minks, foxes, cattle, and rodents (6). PRV infection usually causes severe respiratory/neurological damage to susceptible animals (7). It has placed an enormous burden on the swine industry worldwide (8). Notably, PRV poses a potential risk to the public health as recent studies suggest that rare infection of humans may be possible (9–11). Considering the huge economic losses to pig farming and a possible threat to humans, it is imperative to comprehensively investigate PRV pathogenesis and develop antiviral strategies.

As it has the genome of linear double-stranded (ds) DNA, PRV mainly triggers cGAS-STING axis (12). Nevertheless, certain PRV proteins have been reported to target this signaling pathway for immunosuppression. For example, PRV tegument protein UL21 induces Toll-interacting protein-mediated cGAS autophagic degradation (13). PRV tegument protein UL13 recruits the E3 ligase RING-finger protein 5 to degrade STING in a ubiquitination-dependent manner and directly suppresses IRF3 binding to DNA promoter as well (14, 15). PRV tegument protein UL24 interacts with IRF7 for its proteasome-dependent degradation (16). Despite these findings, the underlying mechanisms by which PRV impedes cGAS-STING axis remain to be fully elucidated.

Non-muscle myosin heavy chain IIA (NMHC-IIA, encoded by *MYH9* gene) is a multifunctional cytoskeleton protein involved in various cellular physiological processes, such as cell movement, cell division, and cell-cell adhesion (17, 18). In addition, several studies report that NMHC-IIA participates in multiple viral infections (19–22). We have recently revealed that NMHC-IIA antagonizes host innate immune responses during RNA virus infections (23). In this report, we uncovered its unappreciated role in dampening DNA virus-induced innate immunity using PRV as a model. NMHC-IIA was shown to inhibit antiviral innate immune responses via suppressing cGAS-mediated signaling during PRV infection. Crucially, NMHC-IIA inhibition by Blebbistatin (BLEB) exhibited the antiviral activity through triggering innate immune responses *in vitro* and *in vivo*.

## RESULTS

### PRV upregulates NMHC-IIA to facilitate viral proliferation

**Fig 1:**
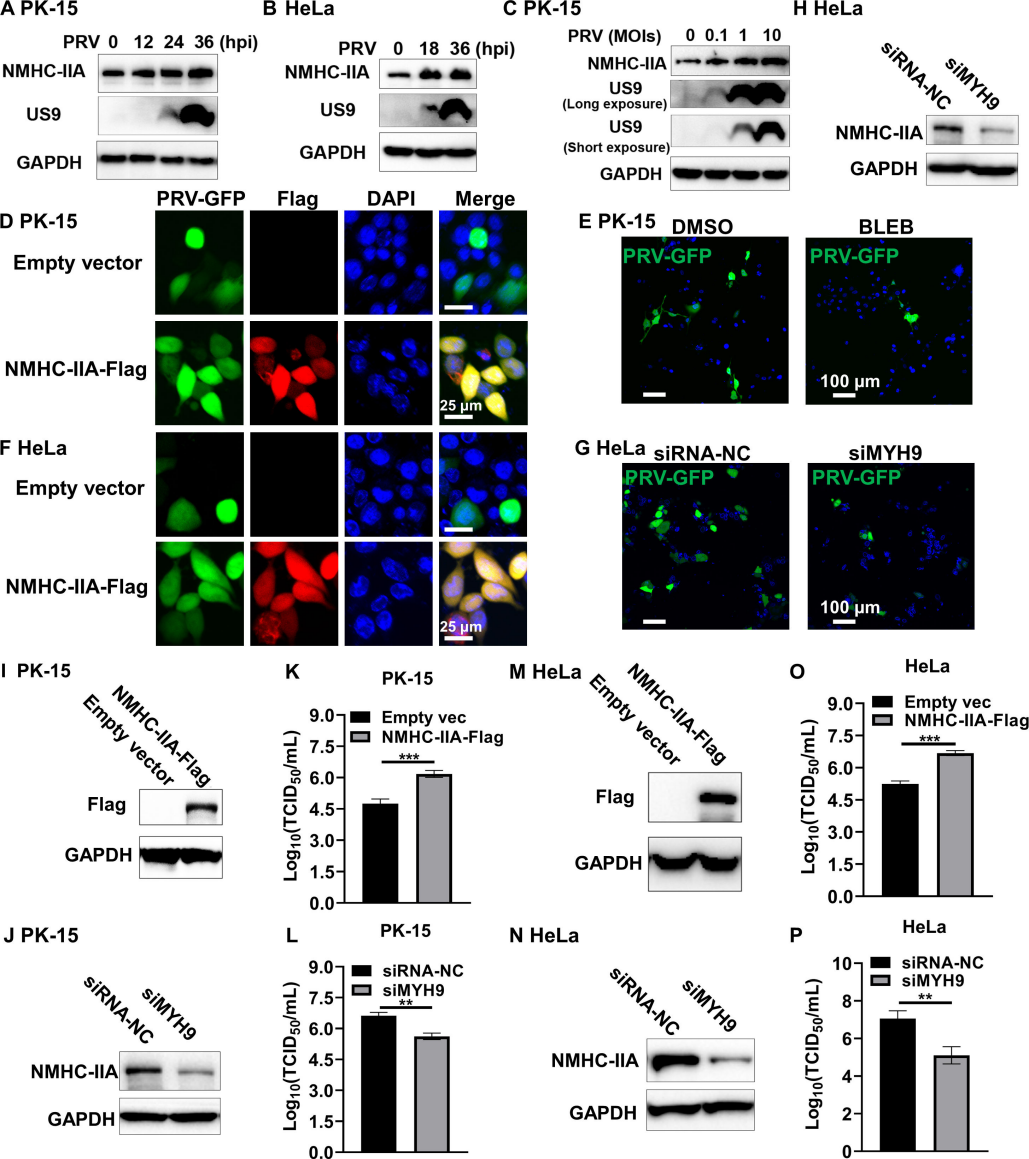
PRV elevates NMHC-IIA to promote viral proliferation. (**A and B**) PK-15 or HeLa cells were infected with PRV (MOI = 1) for indicated time periods. (**C**) PK-15 cells were infected with PRV at different MOIs (0.1, 1, or 10). Expression of the indicated proteins was detected by WB. (**D**) PK-15 cells were transfected with the NMHC-IIA-Flag plasmid for 24 h and then infected with PRV-GFP (MOI = 0.1) for 12 h. Cell nuclei were stained with Hoechst. The PRV-GFP proliferation was observed by confocal microscopy. The scale bar represents 25 µm. (**E**) PK-15 cells were infected with PRV-GFP (MOI = 0.1) for 2 h and then treated with BLEB (5 µM) for 12 h. Cell nuclei were stained with Hoechst. The PRV-GFP proliferation was observed by confocal microscopy. The scale bar represents 100 µm. (**F and G**) HeLa cells were transfected with the NMHC-IIA-Flag plasmid (**F**) or siMYH9 (**G**) for 24 h and infected with PRV-GFP (MOI = 0.1) for 12 h. The PRV-GFP proliferation was observed by confocal microscopy after cell nuclei staining with Hoechst. The scale bar represents 25 or 100 µm. (**H**) HeLa cells were transfected with siRNA-NC or siMYH9 for 24 h and then infected with PRV-GFP (MOI = 0.1) for 12 h. The indicated protein abundance was examined by WB. (**I–P**) PK-15 (**I–L**) or HeLa (**M–P**) cells were transfected with the NMHC-IIA-Flag plasmid or siMYH9 for 12 h and infected with PRV (MOI = 1) for 48 h. PRV titers were detected by assessing TCID_50_. The indicated protein abundance was examined by WB. Quantitation data were shown as mean ± SD from three replicates. Statistical analysis was carried out using Student’s *t* test. **P < 0.01 and ***P < 0.001.

We first examined NMHC-IIA expression in porcine kidney-15 (PK-15) cells and human cervix carcinoma HeLa cells after PRV inoculation for different time periods. The results showed that PRV infection elevated NMHC-IIA abundance in a time-dependent manner (Fig. 1A and B). Furthermore, we found that PRV infection increased NMHC-IIA protein levels in a dose-dependent manner (Fig. 1C), suggesting that NMHC-IIA was involved in the regulation of PRV proliferation. To identify the role of NMHC-IIA in PRV propagation, we transfected the plasmid encoding NMHC-IIA-Flag into PK-15 cells followed by inoculation with the green fluorescent protein (GFP)-incorporated PRV (PRV-GFP) and observed that PRV-GFP proliferation was facilitated in the NMHC-IIA-overexpressed cells (Fig. 1D). On the contrary, NMHC-IIA inhibition by the specific inhibitor BLEB restricted its multiplication (Fig. 1E). We also proved that NMHC-IIA overexpression contributed to PRV-GFP proliferation (Fig. 1F), while *MYH9* knockdown suppressed its propagation in HeLa cells (Fig. 1G and H). To further determine the role of NMHC-IIA in PRV proliferation, we inoculated naïve PRV into the NMHC-IIA-overexpressed or *MYH9*-knockdown cells (Fig. 1I, J, M and N). Fifty percent tissue culture infective dose (TCID_50_) assessment showed that NMHC-IIA overexpression increased the production of PRV progenies (Fig. 1K and O), whereas NMHC-IIA downregulation restrained PRV production (Fig. 1L and P). These results confirm that PRV upregulates NMHC-IIA to facilitate viral proliferation.

### NMHC-IIA inhibits cGAS-STING-mediated immune responses to promote PRV replication

**Fig 2:**
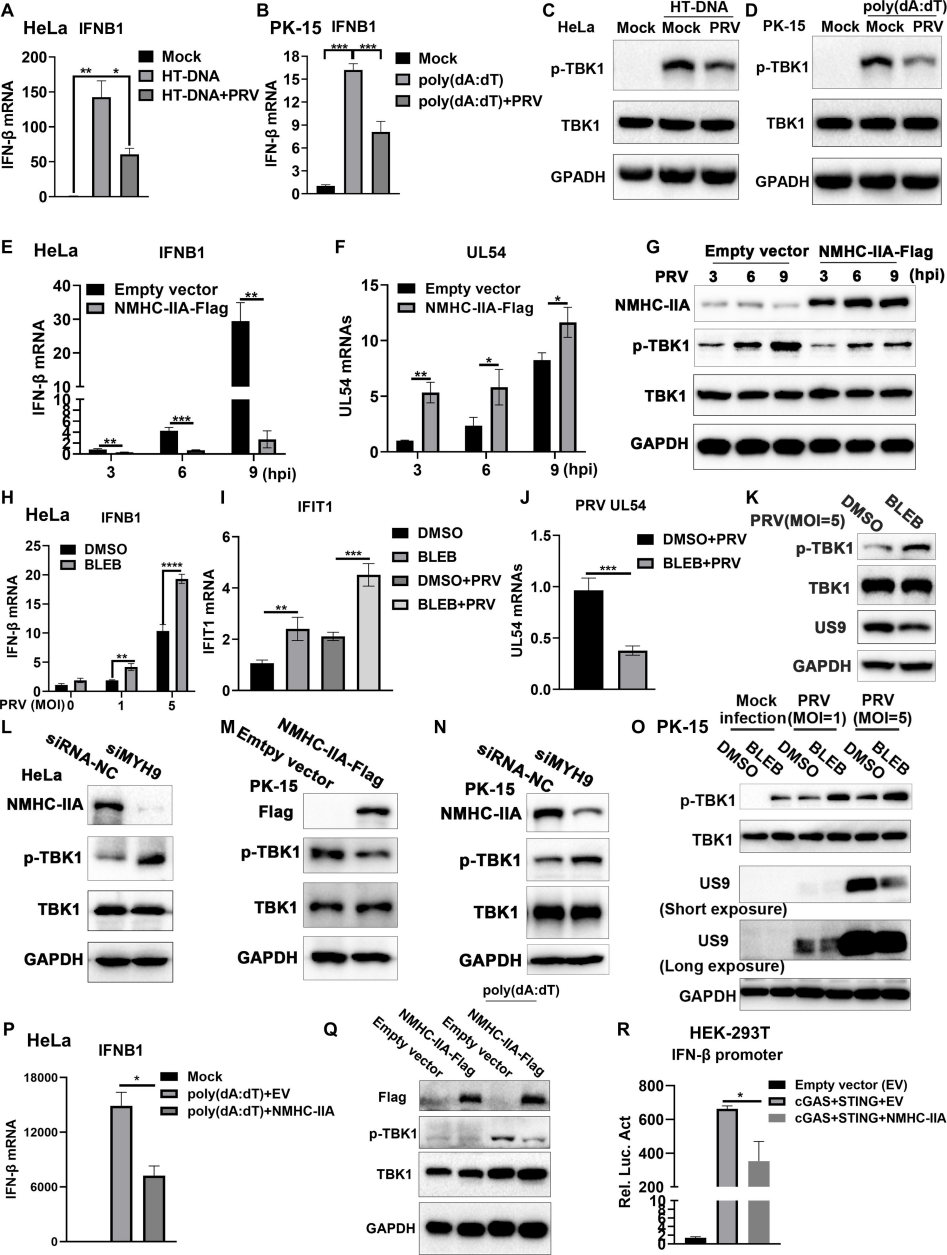
NMHC-IIA antagonizes cGAS-STING-mediated immune responses to promote PRV proliferation. (**A–D**) HeLa or PK-15 cells were infected with PRV (MOI = 1) for 12 h and then transfected with HT-DNA or poly (dA:dT) for 3 h. The cells were collected to detect IFN-β production by RT-qPCR (**A and B**) and the indicated protein abundance by WB detection (**C and D**). (**E–G**) HeLa cells were transfected with the NMHC-IIA-Flag plasmid for 36 h and then infected with PRV (MOI = 1) at 3, 6, and 9 h post-infection. The mRNA levels of IFN-β and UL54 were detected by RT-qPCR (**E and F**). The abundance of the indicated proteins was examined by WB (**G**). (**H–K**) HeLa cells were infected with PRV (MOI = 1 or 5) for 2 h and subsequently treated with BLEB (5 µM) for 12 h. The mRNA levels of IFN-β, IFIT1, and UL54 were detected by RT-qPCR (**H–J**), and the expression of the indicated proteins was evaluated by WB (**K**). (**L**) HeLa cells were transfected with siMYH9 or siRNA-NC for 36 h and then infected with PRV for 12 h. The cells were applied to WB detection. (**M and N**) The NMHC-IIA-overexpressed (**M**) or *MYH9*-knockdown (**N**) PK-15 cells were inoculated with PRV. The indicated protein expression was detected by WB. (**O**) PK-15 cells were inoculated with PRV at MOI of 1 or 5 for 2 h and then treated with BLEB (5 µM) for 12 h. The specific protein abundance was evaluated by WB. (**P and Q**) HeLa cells were transfected with the NMHC-IIA-Flag plasmid or empty vector for 36 h followed by poly (dA:dT) stimulation. The mRNA levels of IFN-β were detected by RT-qPCR (**P**), and the expression of the indicated proteins was evaluated by WB (**Q**). (**R**) Pig IFN-β-Luc reporter plasmid was co-transfected with the plasmids encoding Renilla luciferase, cGAS-HA, STING-HA, and NMHC-IIA-Flag (or empty vector) into HEK-293T cells. The pig IFN-β promoter activation was detected by luciferase assay. Quantitation data were shown as mean ± SD from three replicates. Statistical analysis was carried out using Student’s *t* test. *P < 0.05, **P < 0.01, ***P < 0.001, and ****P < 0.0001.

Subsequently, we explored the mechanisms by which NMHC-IIA contributed to PRV proliferation. Previous studies have shown that NMHC-IIA inhibits innate immunity during RNA virus infections (23), and PRV antagonizes cGAS-STING axis activation (24). Based on these findings, we hypothesized that NMHC-IIA contributed to PRV propagation via antagonizing cGAS-STING-mediated innate immune responses. To verify this hypothesis, we first utilized herring testes (HT)-DNA or B-DNA poly (dA:dT) to activate cGAS-STING axis (25) and detected that PRV infection indeed dampened cGAS-STING-mediated immune responses as indicated by decreased IFN-β production and impaired TBK1 phosphorylation (Fig. 2A through D). We further evaluated the effects of NMHC-IIA on antiviral immune responses during PRV infection and confirmed that NMHC-IIA overexpression suppressed IFN-β mRNA abundance and TBK1 phosphorylation, correspondingly promoting PRV early gene UL54 production (Fig. 2E through G). On the contrary, NMHC-IIA inhibition by BLEB increased the mRNA levels of IFN-β and the ISG-IFN-induced protein with tetratricopeptide repeats 1 (IFIT1) upon PRV infection, while reduced that of PRV UL54 (Fig. 2H through J). Additionally, either BLEB treatment or *MYH9* knockdown elevated the phosphorylation levels of TBK1 in the PRV-infected HeLa cells (Fig. 2K and L), and BLEB treatment reduced PRV small type II membrane protein US9 abundance (Fig. 2K). We also obtained the similar results in PK-15 cells (Fig. 2M through 2O). More importantly, we verified the suppressive role of NMHC-IIA on poly (dA:dT)-induced IFN-β mRNA expression and TBK1 phosphorylation (Fig. 2P and Q). In the meantime, NMHC-IIA overexpression inhibited IFN-β promoter activity upon co-transfection of the hemagglutinin (HA)-tagged cGAS and STING plasmids in human embryonic kidney 293T (HEK-293T) cells, which lack STING expression (26) (Fig. 2R). These results validate that NMHC-IIA functions as an antagonist of cGAS-STING-mediated immune responses during PRV infection.

### NMHC-IIA antagonizes cGAS-STING-mediated immune responses via targeting cGAS

**Fig 3:**
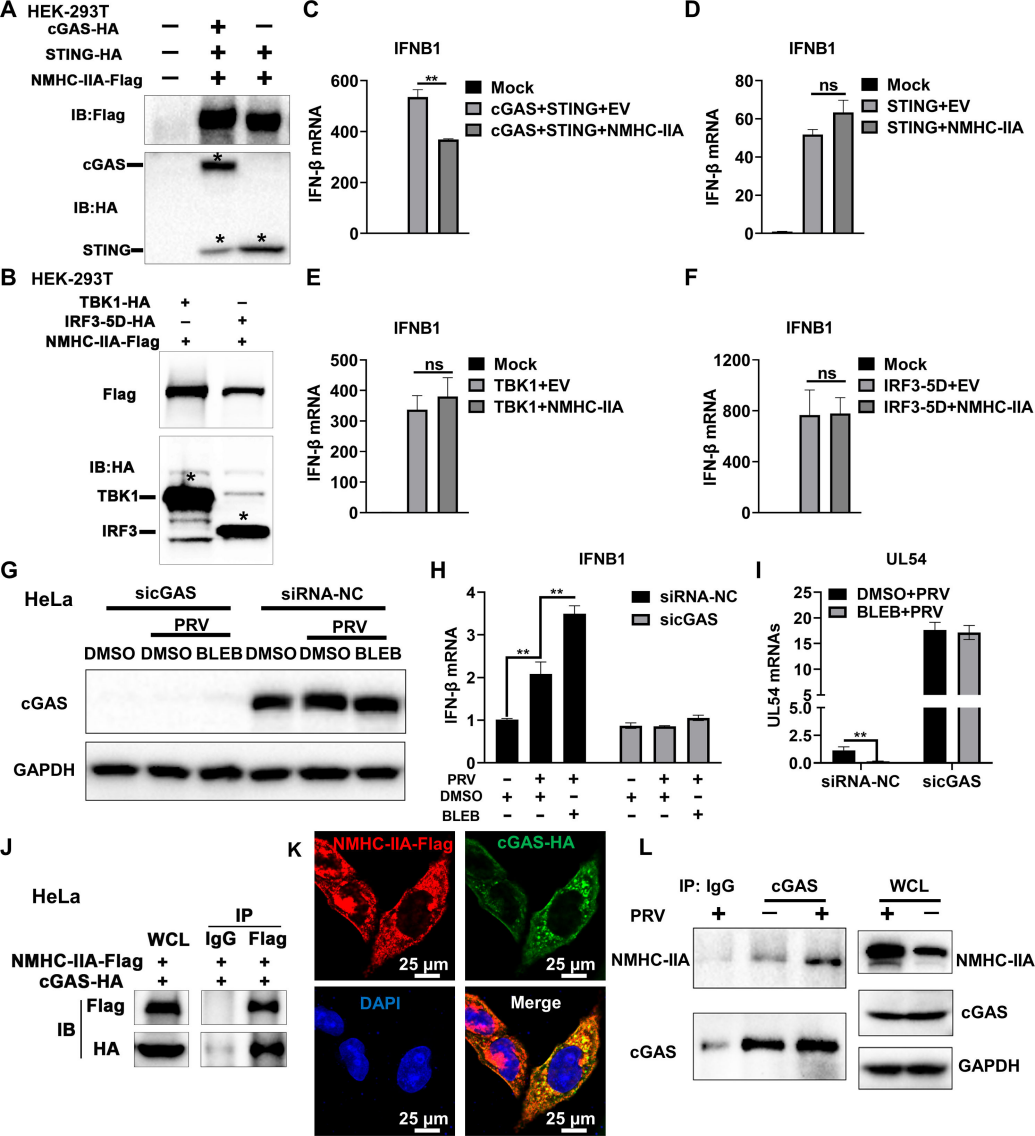
NMHC-IIA targets cGAS to impair its downstream immune responses. (**A–F**) HEK-293T cells were co-transfected with the cGAS-HA and STING-HA plasmids or transfected with the plasmid encoding STING-HA, TBK1-HA, or IRF3-5D-HA for 36 h. The indicated protein abundance was detected by WB. Asterisks marked the target proteins (**A and B**). IFN-β mRNA level was detected by RT-qPCR (**C–F**). (**G–I**) HeLa cells were transfected with siRNA-NC or sicGAS for 36 h followed by PRV inoculation for 12 h. The cells were collected for WB detection of the indicated proteins (**G**) and RT-qPCR detection of IFN-β and UL54 production (**H and I**). (**J**) HeLa cells were co-transfected with the NMHC-IIA-Flag and cGAS-HA plasmids for 48 h and then prepared into WCLs. WCLs were co-incubated with Protein A/G magnetic beads and anti-Flag antibody. The beads were extensively rinsed with ice-cold PBS followed by SDS-PAGE and WB detection. (**K**) HeLa cells were transfected with the NMHC-IIA-Flag and cGAS-HA plasmids for 48 h. The transfected cells were observed by confocal microscopy after nuclear staining with DAPI. cGAS, green; NMHC-IIA, red; nuclei, blue. The scale bar represents 25 µm. (**L**) The PRV- or mock-infected cells were collected for IP and WB detection. Quantitation data were shown as mean ± SD from three replicates. Statistical analysis was carried out using Student’s *t* test. ns, no significance. **P < 0.01.

Since we have proven that NMHC-IIA was a suppressor of cGAS-STING signaling, we attempted to identify the targets of NMHC-IIA in cGAS-STING pathway. We first prepared the HA-tagged cGAS, STING, TBK1, and IRF3-5D (an active form of IRF3) plasmids (27). Each HA-tagged plasmid was co-transfected with the plasmid encoding NMHC-IIA-Flag into HEK-293T cells (Fig. 3A and B). The transfected cells were collected for reverse transcription quantitative real-time PCR (RT-qPCR) detection, and the results showed that NMHC-IIA failed to inhibit STING-, TBK1-, or IRF3-5D-induced IFN-β production (Fig. 3D through F). However, NMHC-IIA suppressed IFN-β mRNA expression after co-transfection of the cGAS and STING plasmids (Fig. 3C), suggesting that NMHC-IIA targeted cGAS to antagonize its downstream IFN-β induction. To further determine that cGAS was the target of NMHC-IIA during PRV infection, we inoculated PRV into the negative control small interfering RNA (siRNA-NC)- or siRNA targeting *cGAS* (sicGAS)-transfected cells for 2 h followed by BLEB treatment (Fig. 3G). As expected, NMHC-IIA inhibition by BLEB significantly elevated IFN-β production and restricted PRV replication (Fig. 3H and I). However, BLEB failed to augment PRV-triggered immune responses and suppressed viral proliferation in the *cGAS-*knockdown cells (Fig. 3H and I). Moreover, we corroborated the exogenous interaction between NMHC-IIA and cGAS in the HeLa cells co-transfected with the plasmids encoding NMHC-IIA-Flag and cGAS-HA by co-immunoprecipitation (IP) and confocal microscopy (Fig. 3J and K). We also detected their endogenous interaction in the PRV-infected cells (Fig. 3L). These results provide evidence that NMHC-IIA inhibit cGAS-STING-mediated antiviral responses via targeting cGAS.

### NMHC-IIA augments the interaction between poly (ADP-ribose) polymerase 1 (PARP1) and cGAS during PRV infection

**Fig 4:**
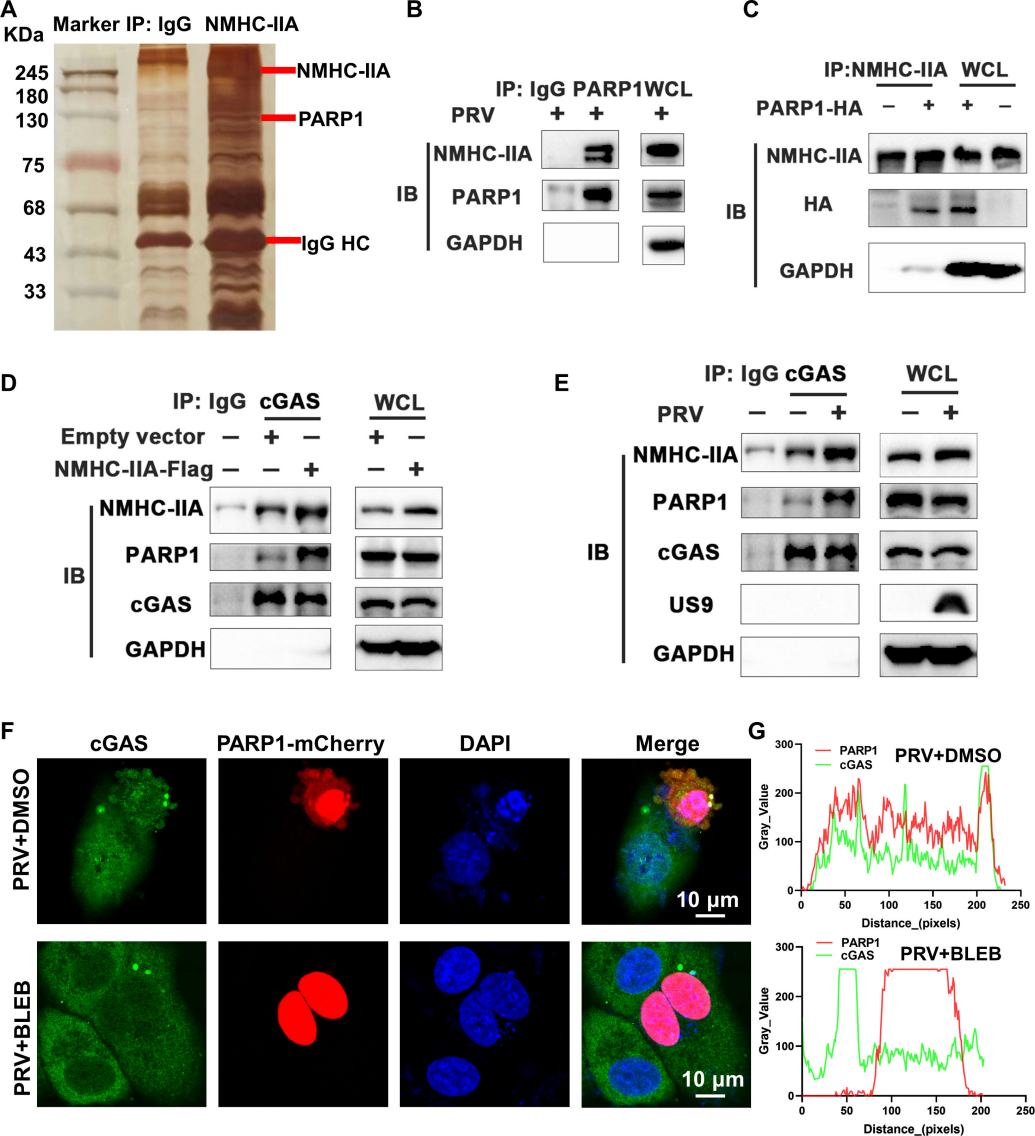
NMHC-IIA enhances the interaction between PARP1 and cGAS during PRV infection. (**A and B**) The PRV-infected HeLa cells were lysed using IP-lysis buffer into WCLs. WCLs were incubated with Protein A/G magnetic beads and anti-NMHC-IIA or PARP1 antibody, where isotype IgG served as a control. After extensive rinse with ice-cold PBS, the beads were subjected to SDS-PAGE using silver staining followed by MS (**A**) or WB detection for the indicated proteins (**B**). (**C**) HEK-293T cells were transfected with the PARP1-HA plasmid for 48 h and then prepared into WCLs. WCLs were subjected to IP assay using NMHC-IIA as the bait. The indicated protein abundance was detected by WB. (**D**) The NMHC-IIA-overexpressed HeLa cells were prepared into WCLs. WCLs were co-incubated with Protein A/G magnetic beads and anti-cGAS antibody. The beads were extensively rinsed with ice-cold PBS followed by SDS-PAGE and WB detection. (**E**) The mock- or PRV-infected HeLa cells were subjected to IP and WB detection of the indicated proteins. (**F and G**) HeLa cells were transfected with the PARP1-mCherry plasmid for 36 h and then inoculated with PRV (MOI = 2) for 2 h followed by DMSO or BLEB treatment for 12 h. Subcellular localizations of cGAS and PARP1 were observed by confocal microscopy after cell nucleus staining by DAPI. cGAS, green; PARP1, red; nuclei, blue. The scale bar represents 10 µm (**F**). Co-localization was analyzed by Image J (**G**).

To further dissect the mechanism by which NMHC-IIA targeted cGAS to suppress cGAS-STING-mediated innate immune responses during PRV infection, we conducted the IP-mass spectrometry (IP-MS) to identify the potential NMHC-IIA-associated proteins. We extracted the whole cell lysates (WCLs) from the PRV-infected cells to incubate with Protein A/G magnetic beads and anti-NMHC-IIA monoclonal antibody or the isotype immunoglobulin G (IgG). The beads were extensively washed and subjected to sodium dodecyl sulfate-polyacrylamide gel electrophoresis (SDS-PAGE). The significantly discrepant band on the SDS-PAGE was applied to MS and identified to be PARP1 (Fig. 4A). We first confirmed the interaction between PARP1 and NMHC-IIA during PRV infection (Fig. 4B). Their interaction was further demonstrated in the HEK-293T cells transfected with the plasmid encoding PARP1-HA (Fig. 4C). PARP1 has been reported to interact with cGAS to antagonize its DNA recognition during herpes simplex virus 1 (HSV-1) infection (28). Considering that NMHC-IIA interacted with cGAS during PRV infection as described above (Fig. 3), we assumed that PRV instigated NMHC-IIA to strengthen the binding of PARP1 to cGAS. We detected that NMHC-IIA overexpression augmented the interaction between PARP1 and cGAS (Fig. 4D). We also found that elevated endogenous NMHC-IIA enhanced their interaction during PRV infection (Fig. 4E). Additionally, we observed the apparent co-localization between PARP1 and cGAS during PRV infection (Fig. 4F and G). In contrast, NMHC-IIA inhibition by BLEB restricted their co-localization (Fig. 4F and G). These results indicate that NMHC-IIA enhances PARP1 binding to cGAS during PRV infection.

### NMHC-IIA enhances PARP1 binding to cGAS for antagonism of its DNA recognition

**Fig 5:**
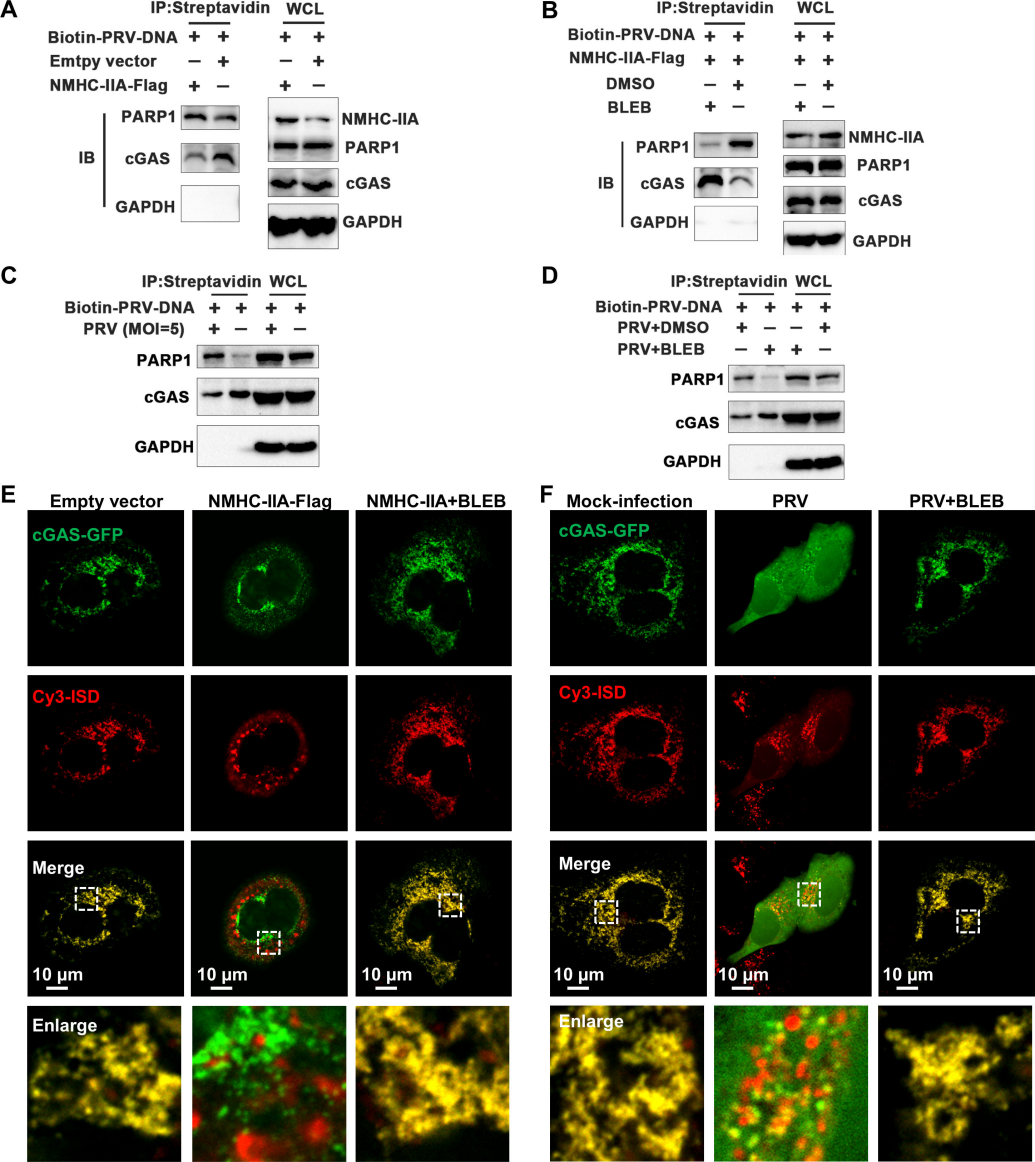
PRV utilizes NMHC-IIA to dampen the DNA-binding ability of cGAS via enhancing its interaction with PARP1. (**A**) HeLa cells were transfected with empty vector or the NMHC-IIA-Flag plasmid for 36 h followed by transfection with biotin-PRV-DNA for 3 h. (**B**) HeLa cells were transfected with the NMHC-IIA-Flag plasmid for 24 h and then treated with DMSO or BLEB for 12 h, followed by transfection with biotin-PRV-DNA for 3 h. (**C**) The mock- or PRV-infected cells were transfected with biotin-PRV-DNA. (**D**) The PRV-infected cells were treated with DMSO or BLEB followed by transfection with biotin-PRV-DNA. Pulldown assay was conducted using streptavidin magnetic beads to capture biotin-PRV-DNA, and its binding proteins were detected by WB. (**E**) HeLa cells were co-transfected with the NMHC-IIA-Flag and cGAS-GFP plasmids for 36 h followed by DMSO or BLEB treatment for 12 h. (**F**) HeLa cells were transfected with the cGAS-GFP plasmid for 24 h and then inoculated with PRV for 2 h, followed by DMSO or BLEB treatment for 12 h. These cells were transfected with ISD-Cy3 for 3 h. Subcellular localizations of cGAS and ISD were observed by confocal microscopy. cGAS, green; ISD, red. The scale bar represents 10 µm.

In the following experiments, we attempted to evaluate the effects of NMHC-IIA-augmented PARP1 binding to cGAS on its DNA recognition. We extracted the PRV DNA, labeled it with biotin (named as biotin-PRV-DNA), and transfected biotin-PRV-DNA into the NMHC-IIA-Flag-overexpressed cells. IP and western blotting (WB) analyses indicated that NMHC-IIA overexpression strengthened the interaction between PARP1 and cGAS, while impeded its binding to biotin-PRV-DNA (Fig. 5A). Conversely, NMHC-IIA inhibition by BLEB recovered cGAS binding to biotin-PRV-DNA with its weakened interaction with PARP1 (Fig. 5B). Consistently, we found that PRV infection impeded cGAS binding to biotin-PRV-DNA (Fig. 5C), whereas BLEB treatment restored the biotin-PRV-DNA binding affinity of cGAS and strikingly lowered its interaction with PARP1 during PRV infection (Fig. 5D). Furthermore, we synthesized and transfected Cy3-labeled interferon stimulatory dsDNA (ISD) into the cGAS-GFP-overexpressed cells, where Cy3-ISD binds to cGAS and induces its activation (29). As expected, NMHC-IIA overexpression dramatically hampered the co-localization of Cy3-ISD and cGAS (Fig. 5E). In addition, compared to the mock-infected cells, we observed that the foci of cGAS were barely co-localized with Cy3-ISD in the PRV-infected cells (Fig. 5F). However, NMHC-IIA inhibition by BLEB treatment recovered their co-localizations in both the NMHC-IIA-overexpressed and PRV-infected cells (Fig. 5E and F). These results demonstrate that NMHC-IIA augments the interaction between PARP1 and cGAS to dampen its DNA sensing during PRV infection.

### NMHC-IIA antagonizes the antiviral immune responses during PRV infection *in vivo*

**Fig 6:**
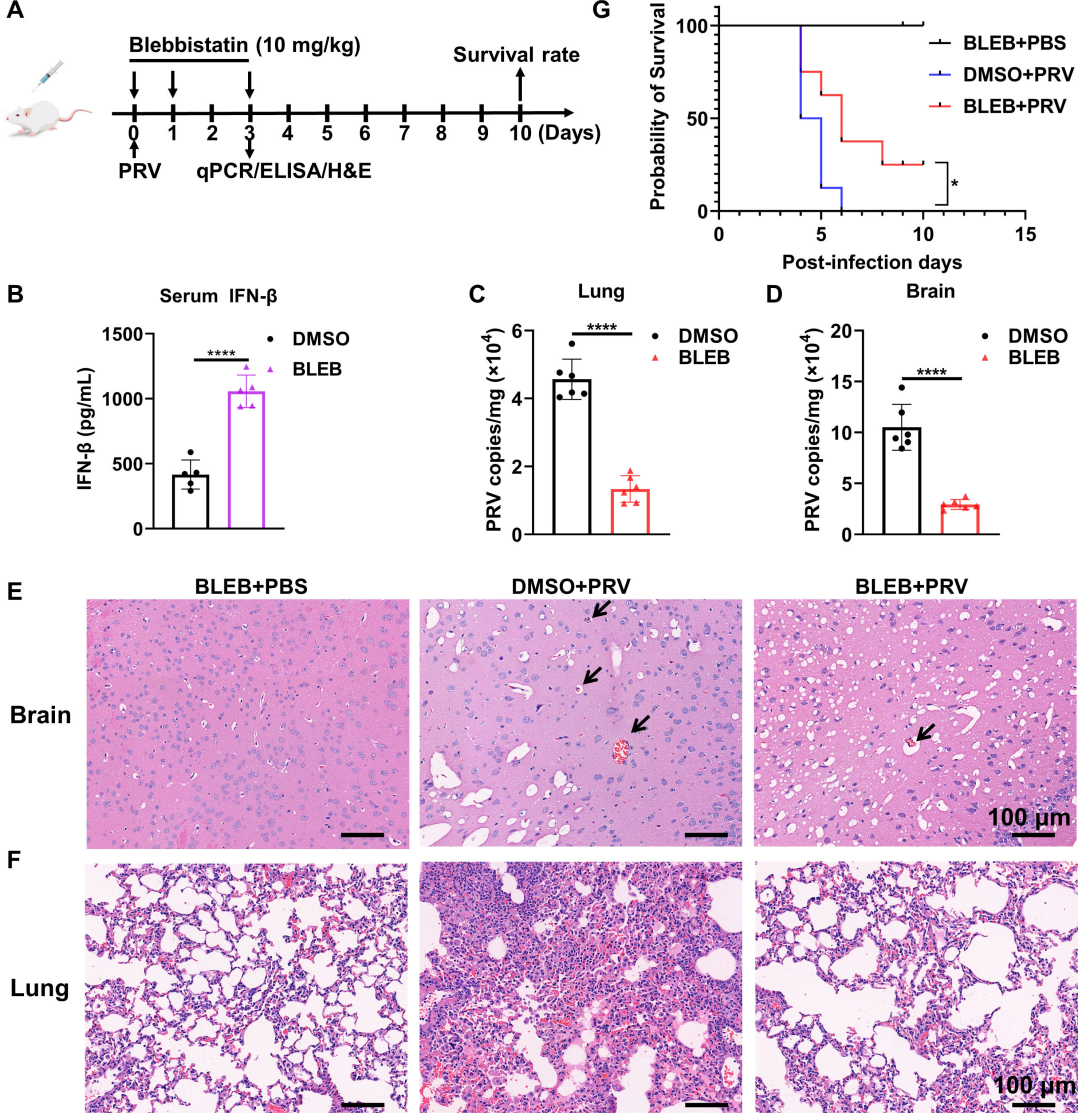
NMHC-IIA inhibition protects against PRV infection via triggering immune responses *in vivo*. (**A**) The scheme for mouse experiment procedure. Female BALB/c mice (*n* = 6 or 8) were intranasally injected with PRV (6 × 10^3^ TCID_50_/mouse) on day 0. The PRV-challenged mice were intraperitoneally injected with DMSO or BLEB (10 mg/kg) on days 0, 1, and 3. (**B–D**) On day 3, mouse sera were collected for enzyme-linked immunosorbent assay detection of IFN-β (*n* = 5) (**B**). PRV genome copy numbers in brains and lungs were evaluated by qPCR for UL54 (*n* = 6) (**C and D**). Statistical analysis was carried out using Student’s *t* test. ****P < 0.0001. (**E and F**) Sections of brains (**E**) and lungs (**F**) were subjected to H&E staining (Lilai biomedicine experiment center, Chengdu, China). Arrows indicate cerebral vascular congestion and hemorrhage in brains. Scale bar, 100 µm. (**G**) The survival rate was monitored daily for 10 days. Statistical analysis was carried out using Log-rank (Mantel-Cox) test. *P < 0.05.

We have proven that NMHC-IIA suppressed innate immune responses to facilitate PRV proliferation *in vitro*. We attempted to evaluate the effects of NMHC-IIA on the antiviral immune responses and PRV proliferation *in vivo*. Unfortunately, we failed to obtain the *MYH9* knockout mice for *in vivo* experiments (data not shown). The loss of *MYH9* was embryonic lethal for mice, consistent with a previous report (30). Therefore, we exploited the NMHC-IIA inhibitor BLEB instead to suppress its function. We inoculated PRV into each BALB/c mice with 6 × 10^3^ TCID_50_ followed by intraperitoneal injection with 10 mg/kg BLEB (Fig. 6A). In line with our *in vitro* data, IFN-β abundance in mouse serum was significantly enhanced after NMHC-IIA inhibition (Fig. 6B), revealing that NMHC-IIA functioned as an antagonist of IFN-I production in the mice challenged with PRV. Furthermore, we collected brains and lungs from the indicated groups to detect the viral loads by qPCR for absolute quantification of UL54 (14, 31). The results showed that NMHC-IIA inhibition significantly reduced PRV replication in these tissues (Fig. 6C and D). Additionally, the PRV-challenged mice exhibited pathological features including cerebral vascular congestion and hemorrhage in brains and interstitial or hemorrhagic pneumonia in lungs, whereas NMHC-IIA inhibition obviously attenuated these injuries caused by PRV infection (Fig. 6E and F). Moreover, we examined the role of NMHC-IIA in mouse survival after PRV inoculation and found that NMHC-IIA inhibition conferred a higher host resistance to PRV infection with a decreased mortality (Fig. 6G). Taken together, these data show that NMHC-IIA plays an immunosuppressive role during PRV infection *in vivo*.

## DISCUSSION

**Fig 7:**
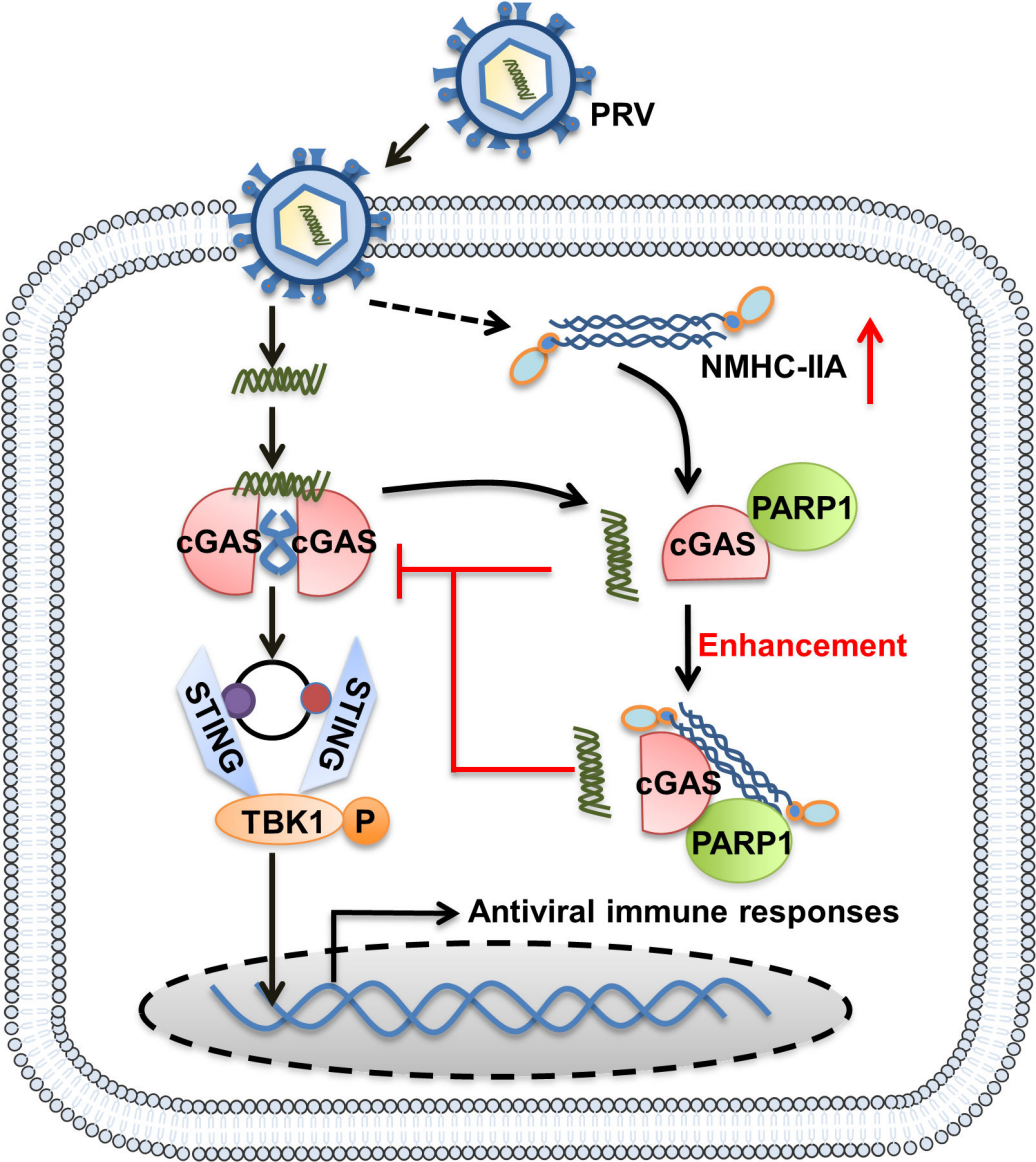
Schematic illustration that NMHC-IIA recruits PARP1 to inhibit cGAS recognition of DNA during PRV infection. PRV infection elevates NMHC-IIA expression, which enhances the interaction between PARP1 and cGAS. The enhanced binding of PARP1 to cGAS subsequently impairs its DNA-binding ability and therefore suppresses the antiviral innate immune responses.

cGAS-STING-mediated immune responses play an indispensable role in combating DNA viruses (32, 33). In order to establish infections, DNA viruses have developed multiple strategies to suppress this axis. Another alphaherpesvirus, HSV-1, has been extensively studied for its interaction with cGAS-STING axis (34–36). For example, HSV-1 directly antagonizes cGAS-STING activation dependent on its proteins, such as the suppression of cGAS enzymatic activity by tegument protein UL37 (36), the counteraction of STING binding to TBK1 by tegument protein UL46 (37), and the blockade of TBK1-triggered STING signalsome by the virion infected cell protein 27 (38). HSV-1 also usurps certain host factors to inhibit cGAS-STING signaling pathway. It has been recently reported that HSV-1 infection upregulates metalloprotease, myb-like, SWIRM, and MPN domains 1 protein (MYSM1) to inhibit STING activation via de-ubiquitinating STING (39). However, there are limited studies concerning PRV-mediated immunosuppression. In addition, most of these studies focus on the interaction between PRV proteins and cGAS-STING axis. To our knowledge, few studies investigate how PRV utilizes host factors for immunosuppression. In this study, we have identified that a host cytoskeleton protein, NMHC-IIA, is upregulated upon PRV infection to promote the interaction between PARP1 and cGAS and impair its recognition of PRV DNA, which thereby antagonizes host antiviral innate immunity for the first time (Fig. 7).

We first found that PRV infection elevated NMHC-IIA expression in a time- and dose-dependent manner, indicating that NMHC-IIA abundance is associated with PRV infection (Fig. 1). Similarly, NMHC-IIA expression is increased in response to an RNA virus-porcine reproductive and respiratory syndrome virus (PRRSV) (19). However, the mechanisms involved in PRV-induced NMCH-IIA upregulation were not yet elucidated, which needs to be explored in the future. Subsequently, we found that NMHC-IIA overexpression facilitated PRV propagation, while *MYH9* knockdown or NMHC-IIA inhibition suppressed its propagation (Fig. 1). A recent report has shown that the high expression of NMHC-IIA in human lungs strikingly facilitates severe acute respiratory syndrome coronavirus 2 infection (22). These data highlight the important role of NMHC-IIA in viral infections. We have previously demonstrated that NMHC-IIA negatively regulates innate immune responses during RNA virus infections (23). Here, we showed that NMHC-IIA inhibited IFN-I production and TBK1 activation upon PRV infection (Fig. 2). These results underline the immunosuppressive function of NMHC-IIA during both DNA and RNA virus infections.

We further determined that NMHC-IIA targeted cGAS to suppress cGAS-STING axis-mediated innate immune responses (Fig. 2 and 3). To dissect the underlying mechanisms, we applied IP-MS and identified that PARP1 was the NMHC-IIA-associated protein during PRV infection (Fig. 4). PARP1 has been shown to interact with and PARylate cGAS to dampen its DNA-binding ability during HSV-1 infection (28). Of note, we found that NMHC-IIA augmented the interaction between PARP1 and cGAS, which impeded its DNA-binding ability after PRV inoculation (Fig. 5). These data demonstrate that NMHC-IIA recruits PARP1 to antagonize cGAS recognition of DNA in response to PRV infection. It is well-established that DNA viruses have diverse strategies to antagonize cGAS-STING-mediated antiviral responses. Additionally, several recent reports have shown that RNA viruses trigger cGAS-STING activation and strategically inhibit this pathway for multiplication (40–43). Therefore, it will be interesting to explore whether this immunosuppressive mechanism of NMHC-IIA is exploited by other DNA viruses as well as RNA viruses. Actually, our previous work reveals that NMHC-IIA recognizes sialic acids on sialylated RNA viruses to antagonize pro-inflammatory responses (23). It will be interesting to explore whether PRV exploits NMHC-IIA to suppress inflammatory responses dependent on sialic acids and identify the sialylated PRV glycoproteins to elucidate their roles in regulation of innate immune responses in future.

Particularly worth mentioning is that the NMHC-IIA inhibitor BLEB is a promising drug candidate for several pre-clinical disease models. For example, BLEB is applied to trigger the potent antitumor immunity (44) and prevent cancer metastasis (45). It has been also utilized to treat neurodegeneration and muscle disease (46). Moreover, BLEB treatment has been reported to provide protection against PRRSV infection in piglets (19). Here, we found that BLEB treatment triggered immune responses and improved the mouse resistance to PRV infection (Fig. 6), supporting BLEB as a potent therapeutic agent for PRV.

Taken together, we have identified that PRV usurps NMHC-IIA to dampen viral DNA recognition by cGAS for antagonism of host antiviral innate immunity. Our results unravel a novel function of NMHC-IIA in inhibiting DNA virus-triggered host immune responses. More importantly, NMHC-IIA has been revealed to exert immunosuppressive effects during DNA and RNA virus infections, which offer it as a potential target for broad-spectrum antiviral strategies.

## MATERIALS AND METHODS

### Cells and viruses

PK-15 (ATCC and CCL-33), HEK-293T (ATCC and CRL-11268), and HeLa cells (ATCC and CRM-CCL-2) were maintained in Dulbecco’s modified Eagle’s medium (DMEM; L110KJ, Basal Media, Shanghai, China) supplemented with heat-inactivated fetal bovine serum (FBS500, Basal Media) and penicillin-streptomycin liquid (100×; P1400, Solarbio, Beijing, China). All cells were cultured at 37°C with 5% CO_2_. The virulent PRV strain HeNLH/2017 (GenBank no. MT775883) and the recombinant strain PRV-GFP were kept in our laboratory (47).

### Antibodies

The specific antibody against cGAS (26416–1-AP), NMHC-IIA (11128–1-AP), NMHC-IIA (60233–1-Ig), Flag (DYKDDDDK) tag (66008–4-Ig), HA tag (66006–2-Ig), glyceraldehyde-3-phosphate dehydrogenase (GAPDH; 60004–1-Ig), PARP1 (13371–1-AP), or β-actin (66009–1-Ig) was purchased from Proteintech (Wuhan, China). The specific antibody against phospho-TBK1/NAK (Ser172) or TBK1/NAK (D1B4) was from Cell Signaling Technology (Danvers, USA). The antibody against PRV US9 was purchased from Developmental Studies Hybridoma Bank (Antibody Registry ID: AB_1553789, Bethesda, USA). Alexa Fluor 488-labeled goat anti-rabbit immunoglobulin (IgG, H + L; A0423) and Alexa Fluor 647-labeled goat anti-mouse IgG (H + L; A0473) were purchased from Beyotime (Shanghai, China).

### Chemical reagents

Protein A/G magnetic beads (HY-K0202) and BLEB (HY-13813) were purchased from MedChemExpress (South Brunswick, USA). BeyoMag streptavidin magnetic beads (P2151), cell lysis buffer for Western and IP (P0013), 4’6-diamidino-2-phenyl-indole (DAPI, C1002), and Hoechst (C1011) were purchased from Beyotime. HT-DNA was purchased from Sigma-Aldrich (D1626, St. Louis, USA). Poly (dA:dT) was purchased from InvivoGen (tlrl-patn, Hong Kong, China). TRIzol reagent (15596018), Lipofectamine 3000 (L3000015), and Lipofectamine RNAiMAX (13778150) were purchased from Thermo Fisher Scientific (Waltham, USA). Phenylmethanesulfonyl fluoride (PMSF, CP8651) and cocktail protease inhibitors (SL1086) were purchased from Coolabor (Beijing, China). 2 × SYBR Green qPCR Master Mix (S2014S) was purchased from Singabio (Tianjin, China).

### RNA interference

siRNA-NC and siRNAs targeting *MYH9* or *cGAS* were synthesized from Bsyntech (Suzhou, China). PK-15 or HeLa cells were transfected with the indicated siRNAs at a final concentration of 50 nM using Lipofectamine RNAiMAX according to the manufacturer’s instructions. The indicated siRNAs were listed in Table 1.

**TABLE 1:** siRNAs in this study

Gene name	Primer (5’−3’)
siRNA-NC	UUCUCCGAACGUGUCACGUTT
siRNA-pig NMHC-IIA	GCAAGCCGCCGAUAAGUAUTT
siRNAs for human NMHC-IIA	GAAGAUCAAUCCAUCUUGUTT/ GCAAGCUGCCGAUAAGUAUTT
siRNAs for human cGAS	CGUGAAGAUUUCUGCACCUTT/ GCAAAAGUUAGGAAGCAACTT

### qPCR assay

Viral DNAs were extracted by TaKaRa MiniBEST Viral RNA/DNA Extraction Kit (9766, TaKaRa, Dalian, China) from the supernatants of PRV-infected cells or PRV progenies. Total RNAs were extracted using TRIzol reagent and then reversely transcribed into cDNAs by the PrimeScript RT Master Mix (RR036B, TaKaRa). Viral genomic DNA was quantified using the UL54-specific primers. The specific cDNAs were detected on a CFX Connect Real-Time PCR Detection System (Bio-Rad, Richmond, USA). The relative mRNA levels were evaluated by the 2^-ΔΔCT^ method using GAPDH as an endogenous control. The indicated primers for qPCR analysis are listed in Table 2.

**TABLE 2:** Primers in this study

Gene name	Primer (5’−3’)
Pig GAPDH F	CCTTCCGTGTCCCTACTGCCAAC
Pig GAPDH R	GACGCCTGCTTCACCACCTTCT
Pig IFN-β F	TTGGCATGTCAGAAGCTCCT
Pig IFN-β R	CTGGAATTGTGGTGGTTGCA
Human GAPDH F	GTCTCCTCTGACTTCAACAGCG
Human GAPDH R	ACCACCCTGTTGCTGTAGCCAA
Human IFN-β F	CTTGGATTCCTACAAAGAAGCAGC
Human IFN-β R	TCCTCCTTCTGGAACTGCTGCA
Human IFIT1 F	GCCTTGCTGAAGTGTGGAGGAA
Human IFIT1 R	ATCCAGGCGATAGGCAGAGATC
UL54 F	TGCAGCTACACCCTCGTCC
UL54 R	TCAAAACAGGTGGTTGCAGTAAA
ISD-F	TACAGATCTACTAGTGATCTATGACTGATCTGTACATGATCTACA
ISD-R	TGTAGATCATGTACAGATCAGTCATAGATCACTAGTAGATCTGTA

### Plasmid construction and transfection

The plasmid encoding human cGAS, TBK1, IRF3-5D, STING fused with HA tag, human NMHC-IIA fused with Flag tag, or pig IFN-β promoter fused with firefly luciferase reporter (pig IFN-β-Luc) was constructed as previously described (23, 48). The plasmid encoding pig NMHC-IIA fused with Flag, human PARP1 fused with HA tag, human PARP1 fused with mCherry, human cGAS fused with EGFP, or human NMHC-IIA fused with mCherry was synthesized from Miaoling Bio (Wuhan, China). These plasmids were individually transfected with Lipofectamine 3000 transfection reagent.

### Virus titration assay

The NMHC-IIA-overexpressed, *MYH9*-knockdown, or BLEB-treated cells were inoculated with PRV (multiplicity of infection, MOI = 1) for 48 h. The supernatants were collected for serial dilution from 10^−1^ to 10^−10^ in DMEM and inoculated into the cells seeded in 96-well plates. The cellular cytopathic effect was counted to calculate TCID_50_ by the Reed-Muench method (49).

### Western blotting

The treated cells were collected for lysis in radioimmunoprecipitation assay (RIPA) lysis buffer (containing PMSF and cocktail protease inhibitors) on ice for 30 min. WCLs were obtained through centrifugation at 13,000 rpm at 4°C for at least 15 min. WCLs were subjected to 10%–15% gradient SDS-PAGE and electro-transferred onto 0.22 m polyvinylidene fluoride membranes (ISEQ00010, Millipore, Chicago, USA). The membranes were blocked in 5% skimmed milk at room temperature (RT) for 90 min and incubated with the specific primary antibodies at 4°C overnight. After washing three times with Tris-buffered saline containing 0.5% Tween 20, the membranes were incubated with horse radish peroxidase-labeled goat anti-rabbit or anti-mouse IgG antibody (A0208 or A0216, Beyotime) at RT for 90 min. The electrochemiluminescence-visualized immunoreactive were imaged using a chemiluminescence imaging system (Bio-Rad).

### IP-MS analysis

HeLa cells were inoculated with PRV (MOI = 2) for 12 h and lysed for IP with the NMHC-IIA primary antibody or isotype control IgG (A7016, Beyotime). NMHC-IIA- or IgG-interacted proteins were eluted and subjected to SDS-PAGE and silver staining. Compared with the bands from IgG-associated proteins, the discrepant bands from NMHC-IIA-associated proteins were cut, digested, and subjected to analysis using matrix-assisted laser desorption ionization-time of flight MS by Shanghai Sangon Biotech Co. Ltd (Shanghai, China).

### Co-IP

The PARP1-HA and/or cGAS-HA-transfected HEK-293T or PRV-infected HeLa cells were harvested and lysed using IP-RIPA lysis buffer with 1 mM PMSF and 1 mM mixture of cocktail protease inhibitors followed by extensive rinse of ice-cold phosphate-buffered saline (PBS). Protein A/G magnetic beads were incubated with the WCLs and the indicated antibody against NMHC-IIA, PARP1, or cGAS at 4°C overnight. The beads were washed two times with RIPA lysis buffer and two times with 0.5% bovine serum albumin (BSA)-PBS using the vortex and then boiled in protein loading buffer for 10 min. The protein samples were subjected to SDS-PAGE and WB assays.

### DNA pulldown assay

PRV DNA was extracted and labeled with biotin using Biotin random prime DNA labeling kit under the manufacturer’s protocol (D3118, Beyotime). Briefly, 1 µg PRV DNA was mixed with Random Primer in Buffer and heated at 100°C for 5 min. The mixture was then added with Biotin-Labeling Mix and Klenow Fragment step by step, which were incubated at 37°C overnight. The PRV-biotin transfected cells were collected and lysed for incubation with BeyoMag Streptavidin Magnetic Beads at 4°C overnight. The beads were boiled in sample buffer after washing with PBS and applied to WB assay.

### Luciferase reporter assay

Luciferase assays were performed in the HEK-293T cells with pig IFN-β-Luc. The pig IFN-β-Luc plasmid (50 ng) were co-transfected with the pRL-TK plasmid encoding Renilla-Luc (100 ng) for normalization of transfection efficiency with Dual-Luciferase Reporter Assay System (E1910, Promega, Madison, USA) in the cells seeded in 24-well tissue culture plate. The NMHC-IIA-Flag plasmid (200 ng) or empty vector (200 ng) was transfected into the cells as well. For luciferase assay, the cells were lysed, and luciferase reporter assay was performed using a dual luciferase reporter assay kit from Promega as described above.

### Cy3-ISD conjunction

A 1 mg/mL solution of ISD (Table 2) was reacted with 25 equiv. of Cy3-NHS ester (C982415, Macklin, Shanghai, China) and 10 equiv. of N, N-Diisopropylethylamine (7087–68-5, Macklin). The reaction was incubated overnight at RT. The reaction mixture was lyophilized and re-dialyzed into H_2_O_2_.

### Confocal microscopy

HeLa cells were transfected with the plasmids encoding PARP1-HA and/or NMHC-IIA-mCherry or PARP1-mCherry and cGAS-HA for 36 h followed by PRV infection or BLEB treatment. The treated cells were washed with ice-cold PBS for three times and then fixed with 4% paraformaldehyde at RT for 20 min. The fixed cells were subjected to the permeabilization with 0.2% Triton X-100 at RT for 5 min. After rinsed with ice-cold PBS for three times, the cells were blocked with 5% BSA at RT for 90 min and incubated with the indicated primary antibodies against HA tag or cGAS at 4°C overnight. The cells were rinsed with PBS four times followed by incubation with the corresponding secondary antibodies at RT for about 60 min. The cells were observed by a confocal laser scanning microscope (Leica TCS SP8, Wetzlar, Germany) after being stained with DAPI or Hoechst at RT for about 10 min.

Cy3-ISD (4 µg) was co-transfected with the cGAS-GFP plasmid (1 µg) in the PRV-infected or NMHC-IIA-Flag-overexpressed cells for the indicated time points. The cells were observed through confocal microscopy after fixation.

### BLEB treatment in the PRV-challenged mice

We performed BLEB treatment in mice according to the method as previously described (47). In brief, 6-week-old BALB/c mice (*n* = 6 or 8) were intranasally infected with PRV on day 0 and then intraperitoneally injected with either dimethyl sulfoxide (DMSO) or BLEB at dose of 10 mg/kg on days 0, 1, and 3 post-challenge. The survival rate was monitored daily for 10 days. On day 3 post-challenge, we collected the lungs and brains for hematoxylin and eosin (H&E) staining (Lilai biomedicine experiment center, Chengdu, China) and viral DNA extraction (*n* = 6) for UL54 qPCR detection, respectively. We also collected mouse sera to detect IFN-β production (*n* = 5) through commercial enzyme-linked immunosorbent assay kit (EK2236, MULTISCIENCES Biotech, Hangzhou, China).

### Statistical analysis

Three replicates were included in all experiments, and each experiment was independently repeated at least twice. All data are presented as group mean and standard deviation (SD). Statistical analyses were performed using GraphPad Prism software (Version 8.0.1, http://www.graphpad-prism.cn/) by unpaired two-tailed Student’s *t* test or Log-rank (Mantel-Cox) test. Asterisks indicate statistical significance as follows: ns, no significance; *, *P*  <  0.05; **, *P*  <  0.01; ***, *P* <  0.001; ****, *P* < 0.0001.

## Data Availability

All data generated or analyzed during this study are included in the article.
